# Exploring Family Perspectives on a Group-Based Hands-on Advanced Carbohydrate Counting Education Program for Children and Adolescents with Type 1 Diabetes: A Qualitative Study

**DOI:** 10.3390/nu16111618

**Published:** 2024-05-25

**Authors:** Zandra Overgaard Pedersen, Sabine Schade Jacobsen, Bettina Ewers, Dan Grabowski

**Affiliations:** 1 Steno Diabetes Center Copenhagen, Department of Diabetes Care, Copenhagen University Hospital—Steno Diabetes Center Copenhagen, Borgmester Ib Juuls Vej 83, 2730 Herlev, Denmark; sabine.schade.jacobsen@regionh.dk (S.S.J.); bettina.ewers@regionh.dk (B.E.); 2 Copenhagen Neuromuscular Center, Department of Neurology, Copenhagen University Hospital, Rigshospitalet, 2100 Copenhagen, Denmark; 3 Steno Diabetes Center Copenhagen, Department of Prevention, Health Promotion and Community Care, Copenhagen University Hospital—Steno Diabetes Center Copenhagen, Borgmester Ib Juuls Vej 83, 2730 Herlev, Denmark; dan.grabowski@regionh.dk

**Keywords:** diabetes, child-centered care, education, family, peer-to-peer, carbohydrate counting, nutrition

## Abstract

The care needs of children and adolescents with type 1 diabetes and their families are frequently approached as if they were identical to those of adults, overlooking the distinct challenges young people may face. It has been stated that children and adolescents often find conventional conversations with diabetes specialists tiresome and unpleasant. The present study focuses on familial experiences of an advanced carbohydrate counting program tailored to children and adolescents. The data encompass semi-structured interviews with families who participated in a group-based child-centered advanced carbohydrate counting program. The analysis revealed five themes: (1) peer-to-peer interaction is an essential determinant of sharing and learning; (2) illness perception significantly influences dietary intake; (3) normalization of diabetes in everyday life eases the disease burden; (4) repetition of dietary knowledge is important for retention; and (5) creating a safe and playful learning environment is crucial to engaging children and adolescents in their own treatment. The present findings suggest that it would be beneficial to explore and consider alternative teaching approaches that are adapted to a more interactive and engaging learning environment that is specifically tailored to children and adolescents. This entails moving away from traditional individual approaches.

## 1. Introduction

The incidence of type 1 diabetes (T1D) in children is increasing [1,2]. T1D is an illness that requires daily vigilance and attentiveness, which involves daily blood glucose monitoring, advanced carbohydrate counting (ACC), and insulin dose estimations, depending on the amount of carbohydrates in the upcoming meal. ACC is an approach used to estimate the bolus insulin dose needed to metabolize the total amount of carbohydrates in the consumed meal [3].

The International Society for Pediatric and Adolescent Diabetes (ISPAD) recommends that ACC be presented at the onset of T1D, along with individualized nutritional education [4]. ACC allows flexibility in carbohydrate intake and improves quality of life and glycemic control in children and adolescents with T1D [4]. It has been documented that macronutrient composition, fiber intake, and precise carbohydrate estimations improve glycemic control [4,5,6,7].

Evidence has also shown that the accuracy of parents’ carbohydrate estimations is associated with lower hemoglobin A1c (HbA1c) in children with T1D [8]. The association between late diabetic complications and increasing HbA1c has been well documented in observational studies [9,10,11]. For children diagnosed with T1D before age seven, achievement of glycemic targets is of particular importance, as such young children have an increased risk of developing diabetes complications throughout their lifespan [6]. Therefore, attaining recommendable HbA1c levels shortly after diagnosis in children and adolescents seems to have notable beneficial effects.

One Swedish cohort study showed that children and adolescents with an HbA1c level ≥70 mmol/mol within the first year after diagnosis had significantly higher HbA1c levels as adults when compared with children and adolescents who achieved an HbA1c level <50 mmol/mol within the first year [12]. Glycemic control tends to be inadequate among children and adolescents, yet notable improvements typically manifest during early adulthood [13]. Findings from the above studies demonstrate that early intervention close to onset is of particular importance.

Moreover, the teaching approach used for this population appears to be of particular importance. One qualitative study has accentuated that children with T1D feel they are discussed in the third person, overlooked by clinicians and parents, and unable to contribute to conversations during visits to diabetes clinics [14,15]. However, children and adolescents with T1D express a desire to be involved in managing their condition [16,17].

For this reason, we endeavored to implement an ACC initiative designed to meet the needs of children and adolescents. Although the empirical evidence on practical hands-on educational methods is limited [18], we suggest that educational programs for this population emphasize diverse learning strategies, encompassing activities, playful learning, and practical experience with food to enhance engagement and enthusiasm in this population. According to the ISPAD, families with children diagnosed with T1D can benefit from interacting [4], and recent studies have shown that if children are active while learning, the learning outcome improves [19]. These considerations were incorporated into the conceptualization of the ACC program.

The present qualitative study aims to investigate the families’ perspectives on participation in a group-based ACC program and to present the findings in a way that can inspire and inform the development of content for future programs.

## 2. Methods

### 2.1. The Advanced Carbohydrate Counting Program

Focus group interviews with children, adolescents, and their parents were performed during the conceptualization process of the ACC program to ensure that the education program addressed challenges that were important to families with T1D. The ACC program consisted of three educational sessions. Each session lasted two hours and consisted of practical hands-on experiences and educational learning activities. The inclusion criteria for the ACC program encompassed children and adolescents with recently diagnosed T1D (≤6 months) and children within the age ranges 6–11 and 12–15 years with HbA1c levels equal to or greater than 58 mmol/mol. The groups were divided by age and consisted of a maximum of six families per group.

Common to all the education sessions was that the children, adolescents, and parents were assigned to two separate teaching kitchens to prepare a meal and count the carbohydrate content of the prepared meals. Children, adolescents, and parents reconvened to engage in joint dining while discussing everyday obstacles related to nutrition and T1D. Through all the education sessions, time was allocated to sharing experiences. Subsequent learning activities for the children were (1) dietary classification, distinguishing between items that contain carbohydrates and those that do not; (2) memory game or quiz involving information and discussion on the dietary recommendations; (3) physically active carbohydrate categorization based on glycemic index; (4) challenging mixed meals with delayed blood glucose response; (5) quiz regarding carbohydrate content in sweets; (6) categorization of solid foods according to their carbohydrate composition (20–40–50 g); (7) preceding holidays, the children illustrated their meals by drawing on a tablecloth, subsequently making carbohydrate calculations using apps and individual “Carb Cards”. The parents discussed challenges associated with ACC, nutritional advice, and everyday life and calculated the carbohydrate content of recipes. Furthermore, there was an integrated session in which the parents experimented with a complex meal at home, recording both carbohydrate intake and insulin aspart use. Subsequently, the participating parents and a clinical dietitian assessed and discussed the blood glucose curves and carbohydrate counting. Details of the ACC program are presented in Table 1. The educational sessions were supervised by two experienced clinical dieticians, who led the activities and were available for questions about daily challenges related to T1D.

### 2.2. Recruitment and Participants

The target groups for the present study were children and adolescents with T1D and their families who had participated in the ACC program. Participants were selected through convenience sampling [20] as the focus of our investigation was on the individuals’ perspectives following their participation in the program. One researcher (Z.O.P.) recruited participants directly after the final session. Upon participating in the qualitative study, informed consent was provided a priori. The inclusion criterion was participation in the ACC program. In total, nine parents and seven children participated in the qualitative interviews, the majority being relatively newly diagnosed. One parent participated without a child due to the child’s low age (2 years). In one family, two parents participated. The children involved in the study ranged from 7 to 14 years and were predominantly girls. A 2-year-old female, whose mother was the sole participant, did not participate in the study. The median duration of diabetes was 127.5 days. The characteristics of the participants are presented in Table 2.

### 2.3. Data Collection

In systematic text condensation, the theoretical frame is based on a descriptive and exploratory approach [21]. A total of eight interviews were conducted. Four interviews were conducted in person immediately after the final session of the ACC program, while the remaining four interviews were conducted online using Teams within a three-week timeframe. The average duration of the interviews was 29.5 min (range 20 to 46 min). The interviews were semi-structured and consisted of open-ended questions to address the participants’ perspectives on the ACC program. The semi-structured interview guide was based on themes previously identified during the initial focus group interviews in the process of developing the ACC program, as described under methods. The interview guide centered around the participants’ course experiences, their understanding of dietary guidelines, and the extent of autonomy afforded to children in managing insulin injections and carbohydrate estimations. Z.O.P. performed all interviews between May and September 2023. Pilot interviews were conducted in May 2023 to evaluate the themes in the interview guide. No new themes could be detected, and the pilot interviews were therefore included in the analysis. No changes were made to the semi-structured interview guide. The Danish National Ethical Committee has been notified about the project. The present qualitative study did not require approval but has been registered (journal number F-23017789). The General Data Protection Regulation approved the study (registration number P-2023-283).

### 2.4. Analysis

There was no pre-defined limitation on the number of interviews, but they continued until data saturation was achieved [22]. The four steps in systematic text condensation were applied to analyze and manage the data derived from the interviews [21]. First, the interviews were listened to by Z.O.P. to gain an overall impression of the participants’ perspectives. Second, the interviews were listened to again more thoroughly by Z.O.P. to identify meaning units relevant to the research question. The contents of the interviews were not transcribed, but the identified meaning units were manually written in an interview log, and meaning-bearing units were transcribed. Third, meaning units were then carefully condensed into labeled codes by Z.O.P. and D.G. The interviews were listened to once again to extract data related to the identified codes. This process was iterative in nature. It involved decontextualization and recontextualization to avoid reductionism and ensure the accuracy of the meaning of the statements. In the final step, to ensure accuracy, the data were synthesized and compared with the original meaning units by Z.O.P. and D.G.

## 3. Results

The analysis revealed five primary themes, which are listed below. Table 3 provides an overview of the themes, which are interdependent and interrelated but represent imperative aspects of the experiences of participating in the ACC program tailored to children and adolescents.

### 3.1. Peer-to-Peer Interaction Is an Essential Determinant of Sharing and Learning

The analysis shows that having the opportunity to be with other individuals and families with T1D is the most important aspect of the ACC program. The possibility of talking to other individuals in a similar situation produced a feeling of relief and reassurance:

*“That means quite a lot mentally, being with others. Togetherness and experiencing things, and it’s really nice to learn something and prepare some food together.” (#2, mother)*

It became clear that one helpful factor in the learning process was learning from other families’ experiences and knowledge. By participating in a peer-to-peer learning environment, with opportunities for peer tutoring, the families achieved insights into areas they had no prior experience with. This was addressed in several interviews:

*“The space, along with other parents and a dietitian, I actually just think it’s that space, you sign up for. The conversations that arise, you can’t really schedule those, they just happen suddenly. Then someone has tried being in the swimming pool, and you haven’t experienced that yourself, those are the things you need to talk about.” (#3, mother)*

Sharing inter-familial experiences while preparing food had a significant impact on the families. The topics of those conversations arose spontaneously and offered learning opportunities. As mentioned in the quote above, many of the conversations were unexpected. These conversations provided insights into what the families can expect in the future:

*“They have slightly older children, so I can see what’s coming. I can see that my daughter is going to be able to participate more than right now, when all the responsibility lies with me and her father.” (#3, mother)*

Throughout all the interviews, the mirroring within the peer group and the relationships among the families were highlighted as one of the most significant elements of the sessions. There were individual differences concerning whether peer support contributed to acquiring new knowledge and changing their habitual behavior. The duration of the individual family’s life with diabetes appeared to influence the extent to which the families facilitated learning and acquired new knowledge:

*“Now, we’d been in this game longer than the others, so we could help them a bit with some of the challenges they were facing.” (#6, mother)*

Several of the informants noted that they had very few friends with whom they could talk about diabetes. They experienced that people in their surroundings could not relate to the challenges they were facing daily, which contributed to the feeling of being alone. The sessions provided an opportunity to meet others struggling with the same challenges, which facilitated an experience of being part of a group and belonging.

*“I feel less alone after the course.” (#1, son)*

The families generally expressed an experience of feeling less alone after the ACC program. The sessions provided a safe space for sharing daily struggles related to diabetes management and an opportunity to share sensitive information about how it feels to live with diabetes, all without any prejudices:

*“I don’t feel alone anymore. Because all the other parents I know or talk to, they have no idea and don’t really understand, but I can hear that they have a lot of prejudices about diabetes. Before the course, I felt quite alone. It provided a sense of networking that I haven’t received from nurses or at the hospital.” (#1, father)*

*“We’ve cooked food, and I’ve made new friends.” (#4, daughter)*

The findings highlight the pivotal role of community and peer support in the experiences of families living with T1D. Central to the program’s impact was that it gave families the opportunity to come together, fostering a sense of togetherness and shared understanding, which emerged as a key mechanism for knowledge acquisition and behavior modification within the program.

### 3.2. Illness Perception Significantly Influences Dietary Intake

It was striking that the families often had a specific perception of the magnitude of the disease burden related to dietary management. The perception that T1D necessitates adherence to a specialized diet may result in feeling marginalized within the family dynamic, as children or adolescents may experience restrictions on their dietary choices proposed by either themselves or their parents. Upon disease onset, families are immediately introduced to CC and insulin estimations, which can lead to an unnecessary emphasis on dietary management, followed by many restrictions, which can be challenging to disengage from. Therefore, it may be beneficial to remind families that the dietary recommendations are the same for the whole family and the rest of the population.

*“I actually thought there were different dietary guidelines when you had diabetes, I thought the guidelines were stricter. That there were the seven dietary guidelines and with diabetes, there was an additional layer of avoiding even more sugar, but that’s not the case, we all have to think that we all have to follow them. So, I think it will bring something good for the whole family, and that’s why it was good to review the seven dietary guidelines again.” (#3, mother)*

This intense focus on food can lead to perceiving food solely as a form of treatment, with a rigorous emphasis on identifying which food items may cause postprandial hyperglycemia and should be abstained from to attain stable postprandial blood glucose levels. This leads to avoidance of certain food items, and when the child encounters them, they may feel uncertain about how to manage their bolus insulin.

*“The course has mediated, so I don’t only see food as treatment; suddenly, giving her food feels like giving her medicine, which the course has made me realize that it isn’t, it’s just food; we just need to get it right.” (#3, mother)*

The findings revealed a significant challenge faced by the families regarding the perceived burden of managing T1D, particularly concerning dietary restrictions. This perception often resulted in a sense of marginalization within the family and in other social dynamics, with children and adolescents feeling constrained by dietary limitations, causing them to unnecessarily avoid specific dietary items.

### 3.3. Normalization of Diabetes in Everyday Life Significantly Eases the Disease Burden

Social gatherings can be experienced as intimidating for both the child and the parents because the macronutrient composition of the food served is unknown. This fear of being unable to handle the food items properly and the glycemic outcome can impact various aspects of daily life. It may cause families to refrain from inviting friends for dinner or cause children to exclude themselves from social gatherings.

*“We’ve gained a bit more courage to try inviting some people over, understand us correctly, we may have held back a bit on making plans because of this new situation, it’s always the first and second arrangement that you have to get through, so it’s given me much more courage to just, if some friends come over to play, fine, we’ll just do that, then we’ll figure out the food, because we have knowledge from the program.” (#1, father)*

It is common for insulin injections, performed in the company of others, to be associated with unease and nervousness. Taking insulin in front of others can be a difficult barrier to overcome. It can be less intimidating when it is undertaken together with other people in the same situation, and this in turn can help them feel comfortable administering insulin in other settings.

*“I’m less afraid of taking insulin, when there are other people there.” (#1, son)*

The experience of normalizing actions can have an impact on other aspects of life.

*“It also normalizes, in a way, the fact that you’re not dealing with it alone, for the children but also for parents who come together and share the experience.” (#8, mother)*

For the families, it was important that their meetings with a dietician did not focus on admonishing their specific food choices but instead helped families see how they could manage bolus insulin in relation to their habitual food preferences without feeling stigmatized.

*“It won’t be in a way where you feel like you’re not healthy enough at home, but it’s much more practical and approachable, without having to feel guilty.” (#8, mother)*

*“I was very positively surprised by your professional competencies, that we could come to you with more than just the food, I know that it’s primarily about the food, but we talked about everyday life. When we normally come inside SDCC, it’s very treatment focused of course, but conversations about everyday things is what you need to land in, and to talk into it. It was really nice that it was brought down to the everyday level, that it wasn’t just food as treatment.” (#3, mother)*

The experience of being immersed with other families with T1D, after feeling alone in managing the complexities of the disease, made an impression on most of the families. Sharing diabetes-related challenges with peers facilitated a feeling of being normal. It also became evident that it was important that the dietitians had adopted a nuanced approach to dietary recommendations and recognized the challenges that individual families faced in their daily lives. By acknowledging and addressing these challenges within the context of everyday life, interventions can effectively promote a more normalized experience.

### 3.4. Repetition of Dietary Knowledge Is Important for Retention

Acquisition of new competencies and knowledge inevitably shapes the overall course experience. The informants mentioned several times that they felt the new information could be assimilated with their pre-existing knowledge. When new behavioral strategies were assimilated into pre-existing schemas, the individuals often found it easier to adopt new knowledge and make behavioral changes because they could relate to the new strategies.

*“It was good to hear it again, because I’d forgotten much of it.” (#2, daughter)*

*“I believe more that I sense it’s been built upon something, already existing knowledge.” (#1, father)*

However, many families experienced that food was the only factor affecting the glycemic response. This misinterpretation often led to frustration, especially in families who had alternated between various strategies for calculating the carbohydrate content in the same meal or experimented with different macronutrient compositions to modify the postprandial blood glucose response. Insights into such factors as the “dawn phenomenon” helped the parents acknowledge that, despite their efforts, the postprandial blood glucose response might be beyond their expertise. This facilitated a feeling of reassurance and showed that their endeavors were adequate. By facilitating group-based learning, the informants had an opportunity to share their beliefs and understanding with peers and a professional. This space facilitated discussions that promoted critical thinking, active engagement, and openness to revising beliefs based on new evidence, encouraging reflections and feedback that mitigate misunderstandings and encourage correct understandings.

*“We’ve talked a lot about breakfast, where we had a perception that we haven’t quite understood it here at home, so it was nice to talk about that it’s not just the food, it’s also the body in the morning and the hormone levels in the morning. There we talked about different breakfasts, and that was quite nice.” (#3, mother)*

*“I don’t have that much understanding of biology either, so I absolutely feel like I gained something there too.” (#7, mother)*

The analysis showed that the conversations and educational elements produced a deeper understanding of different aspects that affect the glycemic response. When the actual glycemic response deviated from the anticipated glycemic response, the informants tended to attribute this solely to inadequate carbohydrate calculations, even though other biological factors could be involved. The children and adolescents highlighted the importance of simplifying the equations. This insight underscores the importance of fostering confidence and making ACC more accessible, which is of particular importance because over time, young people gradually assume greater responsibility for ACC. This was addressed by one of the children:

*“I guess the way I think about how you could calculate stuff, just simplifying it, instead of thinking about taking everything that you have into account, instead you just take the main carbohydrate source, just like the pizza, not counting the pizza sauce and the other stuff.” (#1, son)*

The families reported feeling adequately prepared to confront daily obstacles. They found that an ACC program tailored to children and adolescents both equipped them with essential competencies to navigate and manage their daily obstacles and fostered confidence in tackling difficulties as they arise.

*“I feel that the course has educated me more about daily life, and that was actually what I hoped it would do.” (#3, mother)*

The analysis showed that empowering children and adolescents to independently estimate carbohydrate content and insulin needs, with parental consultation for validation, not only cultivated confidence but also fostered a greater sense of responsibility. One mother highlighted the effectiveness of this approach.

*“It has turned out to work very well, when you eat fast carbohydrates, you take insulin 15 min before, I think you’ve gotten very good control over that.” (#7, mother)*

*“I count the carbohydrates myself more (after the course).” (#2, daughter)*

*“I think it’s fun to weigh things.” (#5, daughter)*

In daily management of diabetes, there is an extensive focus on insulin and obstacles related to insulin. Dietary intake can easily be overlooked. Therefore, it is easy for families to get the impression that the significance of dietary intake in relation to glycemic control is minor. The study’s overarching findings indicated that attending a nutrition-focused course aimed at enhancing glycemic control was valuable, facilitating a transition from only focusing on insulin to a more holistic approach, including dietary aspects.

### 3.5. Creating a Safe and Playful Learning Environment Is Crucial to Engaging Children and Adolescents in Their Own Treatment

It is evident that engaging in an ACC program tailored to young people—a program in which they were active participants and engaged in educational activities—significantly influenced their experience and knowledge acquisition, in contrast to a traditional consultation that is solely delivered on a one-on-one basis.

*“It’s funny. When I usually talk to a dietitian alone, I usually leave halfway because I find it so boring.” (#6, daughter)*

*“However, it’s generally whoever it is, whether it’s a dietitian, doctor, or nurse.” (#6, mother)*

*“Yeah, then I leave, it’s too boring. In the course, I wanted to stay. I wish it had lasted longer” (#6, daughter)*

During the course, the parents emphasized that they have primary responsibility for diabetes care, including estimations of insulin doses and carbohydrate estimations. Transitioning to giving the child more responsibility can be both challenging and a bit frightening for families. Hence, a safe learning place facilitated by dietitians and playful learning can initiate this transition process.

*“It’s a good course and it’s a good way to get the children more involved. It’s a good opportunity to invite them more into the world of carbohydrate counting and to relate to what they eat—So you MUST keep doing it.” (#4, mother)*

*“We played the educational part in and it worked really well with the children.” (Interview 3 parent 12:00)*

*“It was probably my favorite thing. I loved that, it makes me wanna make food more.”*

*“I would love to make pancakes again.” (#1, son)*

Overall, engagement in a family- and group-based intervention tailored to children and adolescents resulted in active involvement of the children in their treatment management, while also facilitating understanding of the disease and insight into ACC.

## 4. Discussion

In the present study, we investigated the perceptions of children, adolescents, and their parents regarding their participation in an ACC program. Five themes were identified: (1) peer-to-peer interaction is an essential determinant of sharing and learning; (2) illness perception significantly influences dietary intake; (3) normalization of diabetes in everyday life eases the disease burden; (4) repetition of dietary knowledge is important for retention; and (5) creating a safe and playful learning environment is crucial to engaging children and adolescents in their own treatment.

Peer-to-peer education creates an opportunity for children, adolescents, and parents to exchange experiences and coping strategies that can facilitate a more nuanced understanding of diabetes management, improve clinical outcomes, and facilitate adherence [23,24]. Moreover, observing their peers successfully manage ACC can motivate children to adhere to ACC, as it makes the method seem achievable and socially acceptable. One study found that one of the motivating factors that helped children with T1D achieve greater self-management in relation to diabetes was engaging independently in activities with peers [25]. The informants consistently referred to this theme in the interviews, and it was further underscored in the results. Families indicated that prior to engaging with the ACC program, they had tended to make ACC unnecessarily complex. Now, however, after having learned from each other and created dialogues in the context of daily obstacles, ACC was simpler. The children learned from each other’s failures and successes.

When a child or adolescent is diagnosed with T1D, it has a profound effect on the entire family. This impact is particularly significant for younger children, who rely on their environment to manage their diabetes and administer insulin doses prior to meals. As the child matures, the responsibilities are progressively handed over from the parents to the child. Therefore, tailoring dietary guidance to children and adolescents is pivotal. A previous qualitative study demonstrated that mathematical calculations regarding carbohydrate content and determining insulin doses were perceived as complex and challenging. These barriers prevented children and adolescents from assuming greater responsibility for managing diabetes [25]. In the present study, simplifying carbohydrate calculations was highlighted as one of the positive insights gained by children and adolescents during the course. Both children, adolescents, and parents reported having achieved a greater understanding of the parameters that impact the blood glucose response. Research has shown that caregivers of children with T1D often experience feelings of isolation when managing the disease [26]. Many of the adult informants in our study described feeling relieved after talking with peers facing similar concerns and obstacles and doing so without experiencing stigma. This underscores the significance of providing a dedicated space for adult caregivers, where they can openly share concerns and challenges with one another.

T1D is a life-changing diagnosis that has an impact on several aspects of daily living. Research has shown that when children receive education and support in ACC and integrate it into their daily lives, this can improve both the children’s and their parent’s quality of life [27]. Additionally, they experience fewer dietary restrictions [27]. The present results support this, as the families indicated that prior to engaging with the ACC program, they had an impression that there were distinct dietary guidelines for individuals with T1D that imposed several restrictions on their daily routines, the goal being to prevent hyperglycemia. Informants also reported feeling confident when participating in social gatherings and hosting friends after participating in the ACC program. This confidence stemmed from their discussions about this topic with peers and practicing insulin dosing for complex meals during the ACC program. This highlights the importance of addressing complex meals, such as pizza, as lack of knowledge in managing them may lead to social isolation. Furthermore, earlier studies have shown that children find traditional conversations with diabetes specialists to be boring and unpleasant [14], indicating that a tailored approach is needed for children.

### 4.1. Methodological Considerations

The families in our study were recruited following their participation in the final course session. This pragmatic approach was chosen so that the families would not need to attend an additional session. However, there is a risk of recruitment bias, as it could be that the families that were satisfied with the course were more likely to sign up for the interviews. This could potentially affect the validity and transferability because all aspects of the construct may not be reflected in the results. Thus, significant elements such as negative experiences of the course might be absent from our analysis and conclusions to some extent.

Predominantly, mothers participated with their children in the ACC program. This limited the representation of fathers’ experiences in the present study, potentially leading to an incomplete reflection of the construct. If more fathers had been interviewed, it might have influenced the dialogue, analysis, and ultimately, the conclusions drawn from the present study.

The determining factor in our study is data saturation, which should be sufficient to illuminate the study’s objective [22]. Having a narrow aim and dense sample specificity results in higher data saturation and therefore requires fewer informants [22]. The objective of the present study is relatively narrow, and consequently, fewer participants are needed to achieve sufficient data saturation.

In seven of the eight participants, estimated HbA1c improved during the course. Positive experiences in the course could be related to notable improvements in glycemic control. However, the families were not advised to monitor their glycemic control; they were only informed that the data would be collected. Therefore, influence in this regard is expected to be relatively minimal, but it cannot be excluded.

In qualitative studies, the researcher’s position is of great importance because the researcher’s attitudes inevitably influence the study. One central methodological challenge in the present study is that the primary researcher, the educator, and the interviewer are the same person, which increases the risk of an echo effect, which refers to when the researcher inadvertently influences the results through the researcher’s own preconceptions, biases, or prior knowledge. To minimize the echo effect, the investigators adopted a reflexive approach to challenge assumptions and reflected on how their background might influence the interpretation of the data. We also employed triangulation and coding independence (Z.O.P. and D.G.) to enhance the validity of our findings and reduce the risk of random error. In this regard, it should be emphasized that D.G. neither served as an educator for the course nor had any other involvement in it. One additional limitation is that one of the educators conducted the interviews with the participants (Z.O.P.). This introduces the potential risk of social desirability bias, as the families had established a relationship with the interviewer, which can affect the dialogue.

### 4.2. Child-Centeredness

There is an increased focus on perceiving children as “beings instead of becomings” and on seeing their perceptions as a source of knowledge [28]. Gaining insights into children’s and adolescents’ perspectives offers profound knowledge. Children perceive their experiences differently from adults and often offer unique interpretations [29,30,31]. Still, interviewing children is often perceived as complex and increases the risk of binary answers that affect the data saturation of the interviews [14]. Consequently, the interviewing techniques employed with adults cannot necessarily be applied to interviews with children. To ensure adequate reflection of children’s perspectives on the construct, semi-structured interviews are often supplemented with drawings or pictures [30]. In our study, we exclusively relied on semi-structured interviews. Consequently, there is a potential risk that fundamental experiences and insights from the child’s perspective might not have been fully captured due to limitations in our interview technique. We were, however, aware of the importance of listening to the children’s insights and did our best to construct the interview guide in a way that invited the children to participate fully in the interviews.

### 4.3. Implications for Carbohydrate Counting Courses Tailored to Children and Adolescents

It has been suggested that if ACC skills are acquired at or near the onset of diabetes, the likelihood of continued utilization of the method by the child or adolescent is maximized [32]. This could be a rationale for tailoring the program specific to the newly diagnosed. This aligns with a statement made by one of the parents, who mentioned that the program is especially suitable for newly diagnosed young people. However, studies have also demonstrated that with increased diabetes duration, there is an increase in carbohydrate estimation errors [33]. This suggests that the course could be continuously offered to children or adolescents who have had their diagnosis longer and have suboptimal glycemic control, the goal being to ensure ongoing reeducation. In our study, informants with longer diabetes durations were underrepresented. Therefore, it would be beneficial for future studies to investigate which content would be most valuable for this population, as their needs may differ from those of newly diagnosed individuals.

### 4.4. Implications for Future Research

There is an increasing focus on personalized treatment for diabetes and other chronic diseases, encompassing both medication and lifestyle interventions [34]. We believe that integrating a child- and adolescent-centered approach is part of providing individualized guidance, which may facilitate the children and adolescents’ active participation in their treatment plan. In addition, this approach may extend beyond T1D and could be applied to other chronic diseases, yielding further research into educational programs tailored to children and adolescents. Our study primarily involved newly diagnosed individuals. Hence, to explore potential refinements in addressing this population’s needs, additional research is required that focuses on children and adolescents with longer diabetes durations.

## 5. Conclusions

In conclusion, our study’s findings suggest that when the healthcare system encounters children with chronic diseases, it should consider employing alternative teaching approaches that engage children more than the traditional one-on-one therapist-patient dialogue.

## Figures and Tables

**Table 1:** Details of the ACC program.

Advanced Carbohydrate Counting Program			
	Day 1	Day 2	Day 3
Children and adults (separate kitchens)	Cooking sessions and ACC	Cooking sessions and ACC	Cooking sessions and ACC
Children	Carbohydrate identification	Categorization of carbohydrates by glycemic index and complex meals	Categorization of solid foods by carbohydrate composition
Children	Dietary guidelines memory game	Quiz on carbohydrate composition found in sweets	Tablecloth drawings and carbohydrate estimations on holidays
Adults	Conversations and teaching regarding dietary guidelines	Recipe calculations	Complex meals in relation to real-time blood glucose curves

**Table 2:** Participant characteristics; HbA1c levels are expressed in millimoles per mole (mmol/mol). The estimated HbA1c levels were determined using continuous glucose monitoring (CGM) data. These estimates were derived from 14-day profiles obtained prior to both the baseline and final session.

Family	Child, Sex and Age	Participating Parent(s)	Duration of Diabetes (Days)	Est. HbA1c Baseline	Est. HbA1c End
1	M12	Father	73	40	46
2	F12	Mother	1832	59	55
3	F2	Mother	91	52	50
4	F7	Mother	131	54	47
5	F6	Mother	308	54	52
6	F9	Mother	424	47	46
7	F14	Mother	124	48	46
8	M8	Mother/Father	68	55	46

**Table 3:** Analytical themes identified from the interviews.

Themes
Peer-to-peer interaction is an essential determinant of sharing and learning
2. Illness perception significantly influences dietary intake 3. Normalization of diabetes in everyday life eases the disease burden
4. Repetition of dietary knowledge is important for retention
5. Creating a safe and playful learning environment is crucial to engaging children and adolescents in their own treatment

## Data Availability

The data presented here are available upon request from the corresponding author. The data are not publicly available due to privacy restrictions.
